# Glutaminase Immunoreactivity and Enzyme Activity Is Increased in the Rat Dorsal Root Ganglion Following Peripheral Inflammation

**DOI:** 10.1155/2012/414697

**Published:** 2011-12-20

**Authors:** Kenneth E. Miller, John C. Balbás, Richard L. Benton, Travis S. Lam, Kristin M. Edwards, Richard M. Kriebel, Ruben Schechter

**Affiliations:** ^1^Department of Anatomy and Cell Biology, Oklahoma State University Center for Health Sciences, Tulsa, OK 74107, USA; ^2^Department of Cell Biology, University of Oklahoma Health Sciences Center, Oklahoma City, OK 73190, USA; ^3^Tulsa Bone & Joint Associates, Tulsa, OK 74146, USA; ^4^Department of Anatomical Sciences & Neurobiology, University of Louisville School of Medicine, Louisville, KY 40202, USA; ^5^Affiliated Dermatology, Scottsdale, AZ 85255, USA; ^6^Department of BioMedical Sciences, Philadelphia College of Osteopathic Medicine, Philadelphia, PA 19131, USA

## Abstract

Following inflammation, primary sensory neurons in the dorsal root ganglion (DRG) alter the production of several proteins. Most DRG neurons are glutamatergic, using glutaminase as the enzyme for glutamate production, but little is known about glutaminase following inflammation. In the present study, adjuvant-induced arthritis (AIA) was produced in rats with complete Freund's adjuvant into the hindpaw. At 7 days of AIA, DRG were examined with glutaminase immunohistochemistry, Western blot immunoreactivity, and enzyme activity. Image analysis revealed that glutaminase was elevated most in small-sized neurons (21%) (*P* < 0.05). Western blot analysis revealed a 19% increase (*P* < 0.05) in total glutaminase and 21% in mitochondrial glutaminase (*P* < 0.05). Glutaminase enzyme activity was elevated 29% (*P* < 0.001) from 2.20 to 2.83 moles/kg/hr. Elevated glutaminase in primary sensory neurons could lead to increased glutamate production in spinal primary afferent terminals contributing to central sensitization or in the peripheral process contributing to peripheral sensitization.

## 1. Introduction

Several animal models of tonic pain, for example, subcutaneous and intraarticular injections of inflammatory agents such as complete Freund's adjuvant (CFA), are used to mimic human chronic pain [1]. During the acute phase of inflammation, bradykinin, serotonin, prostaglandins, ATP, H^+^, and glutamate activate and/or sensitize the afferent limb of primary sensory neurons by increasing spontaneous activity, lowering activation threshold, and increasing or prolonging firing to stimuli [2, 3]. Sensory neurons respond chronically to inflammation by increasing neurotransmitter/neuromodulator, for example, tachykinin (substance P (SP)) and calcitonin gene-related peptide (CGRP), expression and content in dorsal root ganglia (DRG) [4–6], and enhanced immunoreactivity in the spinal dorsal horn [7], skin, and joints [8, 9]. These peptidergic neurons also are glutamatergic [10, 11], using glutaminase (GLS) as the synthetic enzyme for neurotransmitter glutamate production [3, 12]. Despite data regarding functional, morphological, and neuropeptide alterations in sensory neurons, little is known about long-term regulation of glutamate production in tonic pain models.

Acutely, glutamate is released from central primary afferent terminals following noxious stimulation [13–16]. Acute glutamate release, along with SP and CGRP, is responsible for sensitization of spinal neurons leading to persistent or chronic changes [2]. After the induction of knee joint inflammation in monkeys, glutamate-immunoreactive fibers in the spinal cord increase 30% at 4 hr and nearly 40% at 8 hr [17]. At 24 hrs, extracellular levels of spinal glutamate in rats are 150% above controls [15] indicating a possible prolonged, activity-dependent recruitment of glutamate release from central primary afferents. These studies suggest that glutamate production and release is modified during painful conditions.

Alteration in glutamate production at these acute and intermediate time points most likely represents modification in flux control and/or local modifications of glutamine cycle enzymes, such as GLS [18, 19]. Longer-term evaluations of glutamate metabolism have not been performed in tonic pain models as for neuropeptides in DRG neurons. Based on previous glutamate studies and evaluations of neuropeptide production, we hypothesized that persistent inflammation would cause DRG neurons to increase glutaminase production. The present study, therefore, was to examine glutaminase immunoreactivity and enzyme activity in the rat DRG at seven days after adjuvant-induced arthritis (AIA).

## 2. Materials and Methods

### 2.1. Adjuvant-Induced Arthritis

Adult Sprague Dawley male rats, 250–350 g, were used in this study (*n* = 36). Adjuvant-induced arthritis was caused in the right hindpaw (*n* = 20) by the intraplantar injection of 150 *μ*L of complete Freund's adjuvant (CFA; Mycobacterium butyricum, Sigma) emulsified in saline (1 : 1) [20]. Controls (*n* = 16) were naïve rats that received no injection. Procedures in this study were conducted according to guidelines from the International Association for the Study of Pain [21] and the National Institutes of Health publication number 80-23 and were approved by the University of Oklahoma Health Sciences Center and Oklahoma State University Center for Health Science Institutional Animal Care and Use Committees. Efforts were made to minimize the number of animals used for this study.

The L_4_ DRG was examined for the following reason. The tibial nerve innervates the majority of the plantar surface of the rat hindpaw [22, 23]. Approximately 99% of tibial DRG neuronal perikarya of rats are located in the L_4_-L_5_ DRG and the L_4_ DRG contains more than twice the number than L_5_ [22, 24].

### 2.2. Behavioral Testing

Two days prior to and for the days following AIA, rats were tested for pressure sensitivity with von Frey hairs (Semmes-Weinstein monofilaments; Stoelting, Inc.). Rats acclimated for five minutes in a plastic box (25 × 25 × 25 cm) with 6 mm holes spaced every 6 mm [25]. Monofilaments calibrated for specific forces were inserted through the holes underneath to probe the plantar surface of the hindpaw, 5 times in 3-4-seconds intervals in different places on the plantar surface. Filaments with light force were used first, followed by filaments of increasing force. A filament slowly was applied perpendicularly to the plantar surface until bending of the filament occurred. If the paw did not retract three out of five times, the next larger filament was used. The threshold force was defined as the filament (force) that caused the foot retraction without bending the monofilament three out of five times. Using a conversion table for the filaments (Stoelting), thresholds were reported as gram force.

Thermal latencies for the footpaw plantar surface were determined with Plantar Test apparatus (Ugo Basile, Comerio, Italy) at an intensity of 55 mW/cm^2^. Rats were placed on an elevated glass plate (3 mm) in clear plastic boxes with air holes in the lids and acclimated for 10 min. Radiant heat was applied to the plantar surface of the hindpaw and the withdrawal latency recorded. A second test was followed after 5 min. All behavioral testing occurred at 21-22°C with indirect lighting in the testing room.

### 2.3. Glutaminase Immunohistochemistry

For immunohistochemical localization of GLS, rats (*n* = 6 AIA 7-day; *n* = 4 control) were anesthetized with sodium pentobarbital (90 gm/kg) and transcardially perfused with fixative: 0.2% paraformaldehyde (PFA), 70% (v/v) picric acid (PA) in 0.1 M phosphate buffer, pH 7.4 [26]. Initial immunohistochemical studies had indicated that only small-diameter DRG neurons were GLS immunoreactive (IR) [27], but subsequent studies have determined that high concentrations of paraformaldehyde mask antigenic sites on GLS and decrease GLS immunoreactivity [11, 12, 26]. The fixative used in the current study provides a more accurate immunohistochemical staining pattern with all DRG neurons exhibiting GLS immunoreactivity [26]. Right L_4_ DRG were removed and placed overnight in fixative at 4°C; the PFA concentration was increased to 2% for postfixation. DRGs were transferred to 20% sucrose in 0.1 M Sorenson's phosphate buffer, pH 7.4 for 24–96 hr at 4°C. The tissue was frozen, sectioned at 20 *μ*m in a cryostat, thaw mounted onto gelatin-coated slides, and dried for 1 hr at 37°C. Every fifth section was used to reduce the possibility of evaluating a neuron twice. Sections were washed three times for 10 min in phosphate buffered saline (PBS) and incubated in 10% normal goat serum, 10% normal horse serum, 10% fetal bovine serum, 2% BSA, and 1% polyvinylpyrolidone in PBS with 0.3% Triton (PBS-T).

Sections were incubated for 48 hrs at 4°C in rabbit antiglutaminase (1 : 6000; gift from Dr. N. Curthoys, Colorado State University, Fort Collins, Colo) in PBS-T. The tissue was washed three times in PBS and incubated in biotinylated goat anti-rabbit IgG secondary antibody (3 *μ*g/mL; Vector) in PBS-T for 1 hr at room temperature. Sections were washed two times in PBS following secondary antibody incubation, washed in sodium carbonate buffered saline (SCBS), pH 8.5, incubated in fluorescein-avidin (1.5 *μ*g/mL; Vector) in SCBS for 1 hr, and washed three times in PBS. Coverslips were apposed with Vectashield mounting media (Vector) to retard fading of immunofluorescence. Glutaminase purified from rat brain was incubated with rabbit antiglutaminase for an absorption control. Tissue sections incubated with absorbed primary antibody were processed as described above. Other controls included exclusion of primary and secondary antisera.

Immunofluorescent sections of 7 day AIA and control DRG were observed with an Olympus Provis AX70 microscope with a 20x objective and digital images were obtained with a SPOT CCD camera (Diagnostic Instruments). The entire section of DRG was photographed in a series of images and images were saved as uncompressed TIFF files. The exposure time for all images was the same for all tissue sections from all animals. The exposure time was determined empirically so that weakly stained neurons could be distinguished for tracing, but that intensely stained neurons were not oversaturated [26, 28]. This approach allowed images to be evaluated along the linear aspect of immunofluorescence intensity [28]. The glutaminase-immunoreactive DRG images were analyzed using the SCION Image program (Scion Co., Frederick, Md). Only DRG neurons with a nucleus were evaluated. Individual DRG neurons were circumscribed and the area, pixel number, and intensity were recorded. Neuronal cell bodies in the DRG were distributed into the following three sizes for analysis: 100–600 *μ*m^2^ (small), 600–1200 *μ*m^2^ (medium), and >1200 *μ*m^2^ (large) [29].

### 2.4. Glutaminase Enzyme Assay

For GLS enzyme assays, rats (*n* = 6 AIA; *n* = 4 control) were anesthetized (sodium pentobarbital, 90 mg/kg) and decaptitated. Right L_4_ DRG were removed quickly, placed in embedding molds with M-1 mounting media (Lipshaw), and frozen on dry ice. Individual DRGs were sectioned at −20°C on a cryostat at 30 *μ*m, sections were placed in aluminum racks for lyophilization, and samples were stored under vacuum at −20°C. The embedding media was removed from around the lyophilized DRG sections using a Wild Heerbrugg 181300 dissecting microscope and DRG sections were weighed using quartz-fiber balances [30–32].

GLS enzyme assay was performed according to Curthoys and Lowry [33]. Five to six randomly selected DRG sections from rats with AIA and from control rats were placed individually in a 40 *μ*L volume of reaction mixture containing 20 mM glutamine, 100 mM K_2_HPO_4_, 0.6 mM EDTA, 0.01% Triton-X 100, 0.01% BSA in 50 mM TRIS, pH 8.65, for 45 minutes at 37°C. The reaction was stopped by adding 20 *μ*L of 0.7 N HCl and placing the samples at 4°C. Indicator buffer (1 mL) containing 300 *μ*M ADP, 360 *μ*M NAD, 50 *μ*g/mL glutamate dehydrogenase (GDH, rat liver, Boehringer Mannheim, Indianapolis, Ind) in 50 mM TRIS, pH 8.5 was added for 20 minutes, r.t. In this reaction, glutamate produced by GLS is converted to 2-oxoglutarate via GDH with the formation of NADH. Reduction of NAD^+^ was measured using a fluorometer (Farrand Inc.) with an excitation wavelength of 365 nm and emission at 340 nm. Quantitation was accomplished by reacting increasing concentrations of glutamate standards in the indication reaction. The GLS activity from each DRG section was determined and a mean activity for each DRG was calculated.

### 2.5. Western Blot for Total Glutaminase

For immunoblotting, rats (*n* = 5, AIA, control) were killed with CO_2_ and decapitated. Right L_4_ DRG were removed rapidly and homogenized [34, 35]. Whole brain, spinal cord and kidney also were obtained for evaluation. DRG were homogenized individually with lysis buffer (50 mM Tris pH 7.4, 2 mM EDTA, 0.05% Triton-X 100) with phosphatase inhibitor cocktail I and II and protease inhibitor (Sigma). Homogenates were centrifuged (70,000 rpm, 20 minutes) and the protein concentration of the supernatant was measured (BCA Protein Assay Kit, Pierce, Rockford, Ill) to normalize the samples. Rabbit anti-GLS antibody (gift from Dr. N. Curthoys) was bound to M-280 Dynabeads (Invitrogen) conjugated with sheep anti-rabbit antibody [34]. Equal amounts of total protein (75 mg/mL) were exposed to rabbit anti-GLS antibody beads (16 hr, 4°C) for GLS purification [35]. Samples were exposed to a magnet to collect the bead antibody-protein complex. The purified protein was eluted using Laemmli buffer (10 mM Tris, 1 mM EDTA, 2.5% SDS, 5%  *β*-mercaptoethanol, 5% bromophenol blue, pH 8.0) and by heating the samples at 100°C for three minutes.

GLS electrophoresis was performed on a 12.5% homogenous polyacrylamide gel (Phast-System, Promega) [34, 35] along with molecular weight standards (Novagen). Proteins were transferred to a nitrocellulose membrane in a buffer of 25 mM Tris, pH 8.0, 192 mM glycine and 20% methanol at 25 mA for 20 minutes. Immunoblotting was performed using the Protoblot II AP System (Promega) [34, 35]. Membranes were dried at 37°C, rinsed in 20 mM Tris-HCL, 150 mM NaCl, and 0.05% Tween 20, pH 7.5 (TBST), washed in 1% bovine serum albumin (BSA) in TBST, and incubated in rabbit anti-GLS antibody (1/1000, TBST) for 1 hour at room temperature. Samples were washed in TBST followed by incubation in alkaline phosphatase conjugated goat anti-rabbit antibody for 30 minutes. Samples were washed twice in TBST and TBS. Membranes were incubated in Western Blue stabilized substrate for alkaline phosphatase (Promega; 5-bromo-4-chloro-3-indolyl-phosphate, nitro blue tetrazolium).

### 2.6. Mitochondria Isolation for Glutaminase Western Blots

For mitochondrial isolation, rats (*n* = 3, AIA, control) were killed with CO_2_ and decapitated. Right L_4_ DRG were removed rapidly, manually homogenized in a buffer containing 10 mM Tris-HCL pH 7.4, 0.32 M sucrose, 1 mM EDTA. The supernatant was centrifuged and supernatant (P1) use for mitochondria isolation. P1 was exposed to a rabbit ant-porin antibody (Millipore) overnight at 4°C. Samples were exposed to a goat anti-rabbit antibody conjugated to M-500 magnetic beads (Dynal) 30 minutes [36], exposed to a magnet and reconstituted in a Lysis buffer of 50 mM Tris-HCL pH 7.4, 2 mM EDTA, and 50 *μ*L Triton X-100. The mitochondria were mechanically separated from the beads with a Pasteur pipette, the beads removed by a magnet, and the supernatant (P2; rich in mitochondria) was removed. Isolated mitochondria (P3) were broken (P4) by freeze fraction and sonication and a protein assay was performed on the samples. A normalized concentration of total protein was used to purify GLS from the mitochondria homogenate as described above. Gel electrophoresis was performed using 12% gel and separated employing the PhastSystem. Western blots were performed as described earlier.

Digitized images (600 dpi) of the Western blots (total and mitochondrial) were analyzed with Image Tool (UTHSCSA) to quantify the intensities of GLS bands. Digitized images were converted to grayscale, inverted, and a shadow north filter applied to enhance the contrast between the band and background. Bands in each sample were traced separately with an interactive pen on a Cintiq 21UX (Wacom) tablet. Each band was traced three times to reduce bias and the mean calculated for each band.

### 2.7. Statistics

Data from the analyses are reported as mean value with standard error of the mean. A Student's *t*-test was used to determine differences between AIA and control groups (Prism version 5.01, GraphPad Software Inc., LaJolla, Calif). In all analyses, *P* values less than 0.05 were considered significant.

## 3. Results

Rats developed inflammation in the right hindpaw with redness and edema similar to previous descriptions [1]. Nociceptive responses to normally nonnociceptive pressures (allodynia) and decreased paw withdrawal latencies to thermal stimuli (hyperalgesia) were observed in the right hindpaw from rats with AIA (Table 1).

**Figure 1:**
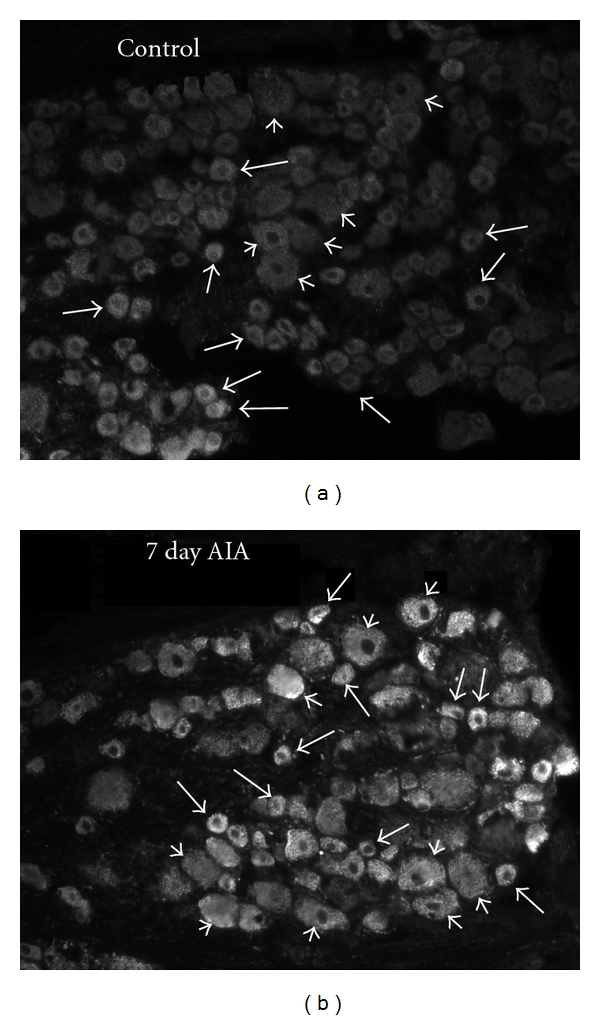
Glutaminase (GLS) immunoreactivity (ir) in rat L_4_ dorsal root ganglia (DRG) following 7 days of adjuvant-induced arthritis (AIA) in the hindpaw. DRG sections were processed simultaneously with a rabbit polyclonal GLS antiserum and photographed under identical conditions. (a) In control sections, GLS-IR was light to moderate in all neuronal cell sizes, small (long arrows), and medium to large (short arrows). (b) Elevated GLS-ir in small (long arrows) and medium to large (short arrows) neurons occurred in the DRG following AIA.

**Figure 2:**
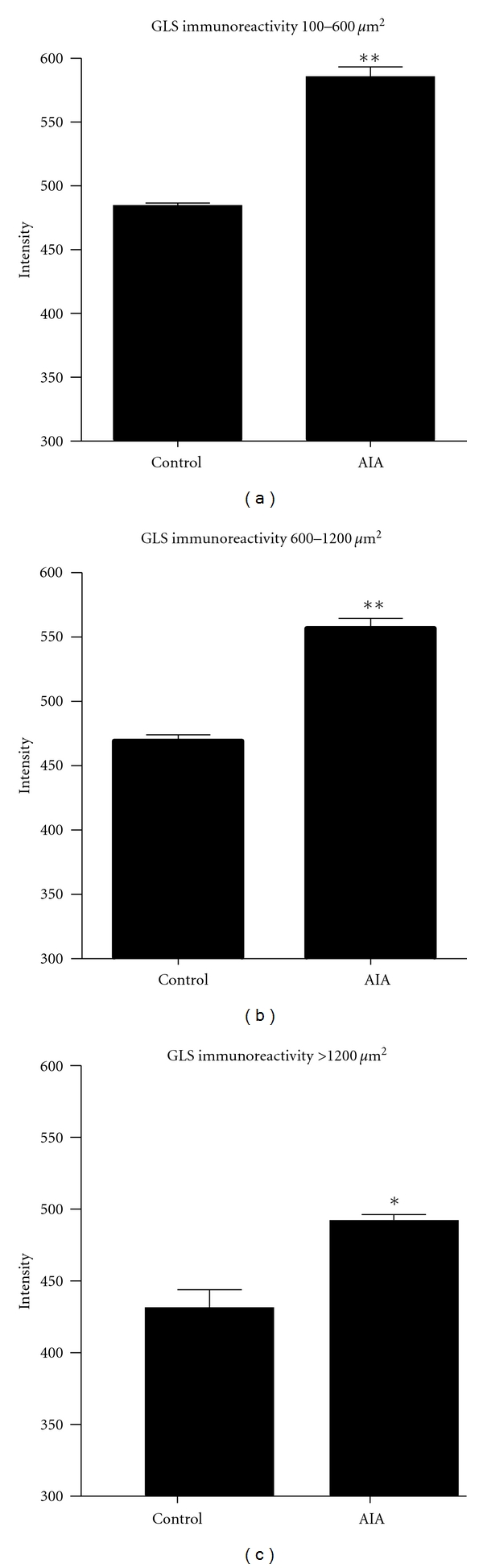
Image analysis of glutaminase (GLS) immunoreactivity (IR) in L_4_ DRG neurons after 7 days of AIA in the hindpaw. Data are presented as intensity divided by the area of the cell. DRG neurons were categorized into three area size groups: (a) small: 100–600 *μ*m^2^, (b) medium: 600–1200 *μ*m^2^, (c) large: >1200 *μ*m^2^. (a) Small-sized neurons in DRG from AIA rats contained a significantly greater GLS immunoreactive signal (**P < 0.01) than controls. (b) Medium-sized neurons in DRG from AIA rats contained a significantly greater immunoreactive signal (**P < 0.01) than controls. (c) Large-sized neurons in DRG from AIA rats were more intensely stained than controls (*P < 0.05).

At 7 days, GLS-immunoreactivity in L_4_ DRG neurons from AIA rats was increased over the control DRG neurons (Figure 1). The GLS-IR intensities of three different sizes of DRG cell bodies, therefore, were analyzed (Figure 2). The overall GLS-IR intensity of small (<600 *μ*m^2^) L_4_ DRG cell bodies (Figure 2(a)) from the AIA rats (585.6 ± 7.7/*μ*m^2^) was greater (*P* < 0.01) than controls (484.6 ± 2.0/*μ*m^2^). This represented a 21% increase in small-sized DRG neurons at 7 days AIA compared to control. The GLS-IR intensity of medium-sized (600–1200 *μ*m^2^) L_4_ DRG cell bodies (Figure 2(b)) from the AIA rats (556.9 ± 7.7/*μ*m^2^) was greater (*P* < 0.05) than controls (469.3 ± 4.9/*μ*m^2^). This represented a 19% increase in medium-sized L_4_ DRG neurons at 7 days AIA compared to control. The GLS-IR intensity of large (>1200 *μ*m^2^) L_4_ DRG cell bodies (Figure 2(c)) from the AIA rats (491.0 ± 5.8/*μ*m^2^) was greater (*P* < 0.001) than controls (431.6 ± 12.2/*μ*m^2^). This represented a 14% increase in large-sized DRG neurons at 7 days AIA compared to control. 

**Figure 3:**
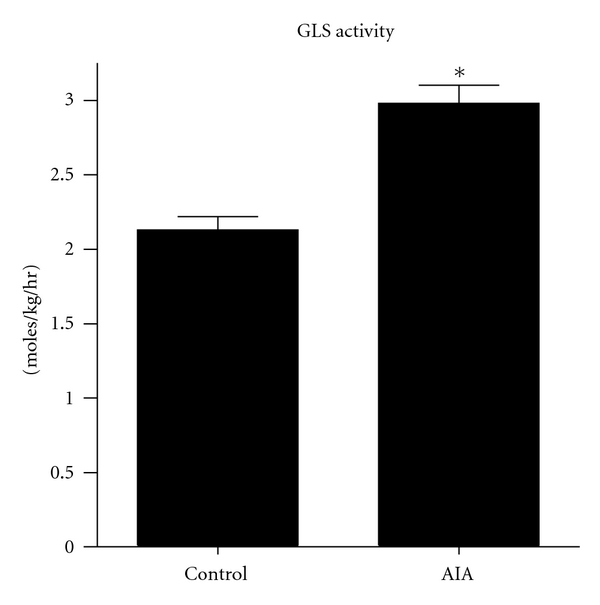
Glutaminase (GLS) enzyme activity in the L_4_ DRG at 7 days AIA in the right hindpaw. GLS activity from the DRG of AIA rats (2.83 ± 0.30 moles/kg/hr) was elevated (*P < 0.05) over control values (2.20 ± 0.18 moles/kg/hr).

Increased GLS enzyme activity in L_4_ DRG's was observed in AIA rats compared to controls (Figure 3). There was a 29% increase in GLS enzyme activity from the AIA compared to control DRG. The GLS activity of the AIA rats (2.83 ± 0.30 moles/kg/hr) was greater (*P* < 0.05) than controls (2.20 ± 0.18 moles/kg/hr).

**Figure 4:**
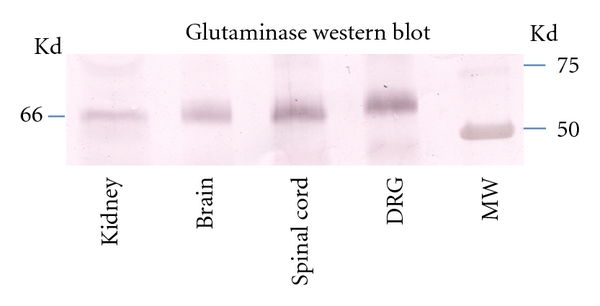
This figure represents the Western blots of glutaminase within the DRG, brain, spinal cord, and kidney. A characteristic 65 kDa band was visualized in all the samples confirming the specificity of the antibody and the presence of the kidney/brain glutaminase isoform within the spinal cord and DRG. Western blots have been cropped to exclude the nonspecific IgG 53 kDa band and enhance the presentation of the 65 kDa band.

**Figure 5:**
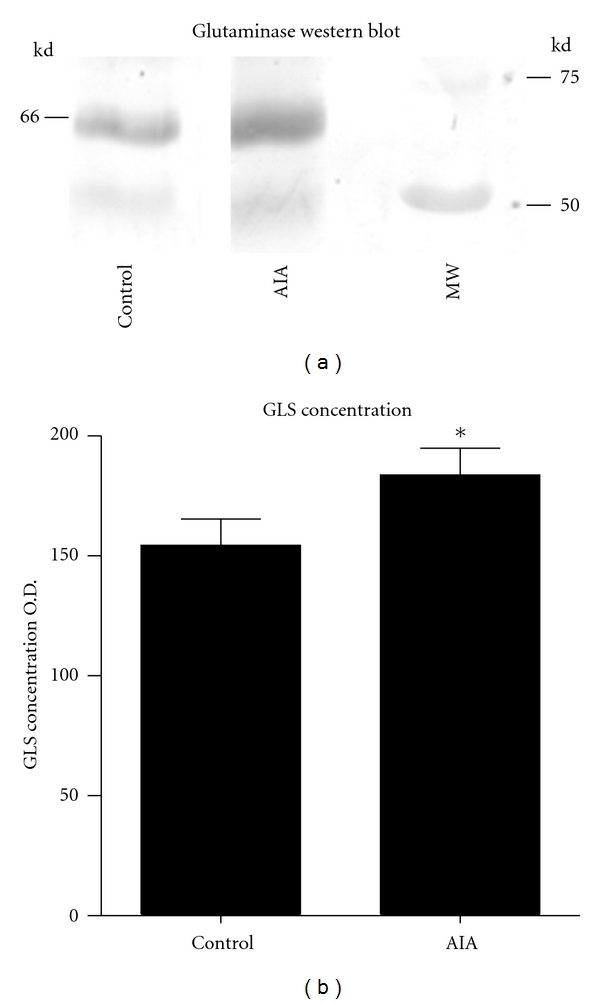
This figure represents the Western blot studies of glutaminase (GLS) within the L_4_ DRG from AIA and control rats. (a) Western blot represents GLS immunoreaction (65 kDa) from the L_4_ DRG of AIA and control rats. Note the increase of GLS immunoreaction within the AIA animals. (b) This graph represents the statistical analysis of the GLS immunoreactivity between AIA and control rats. A significant increase (*P < 0.05) was found between the AIA rats when compared to controls.

**Figure 6:**
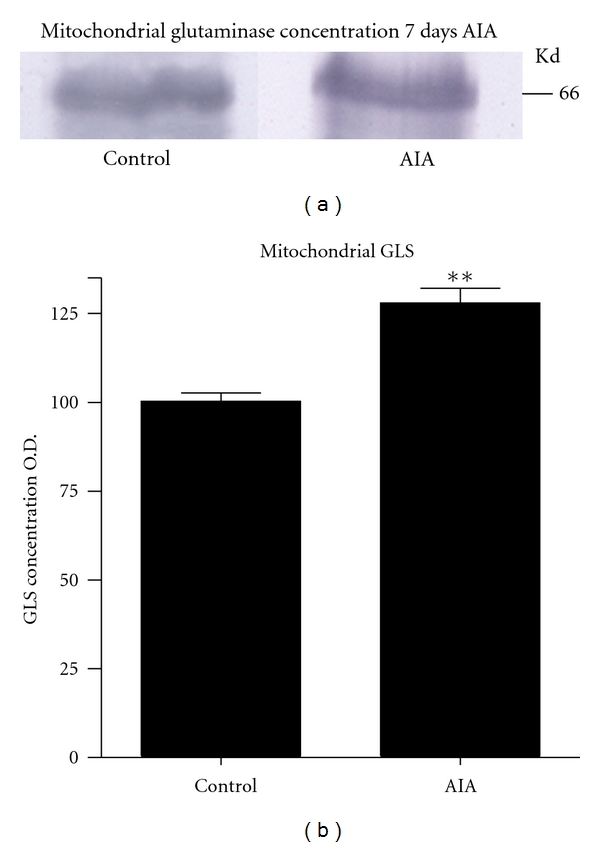
These Western blots represent the mitochondrial glutaminase (GLS) immunoreaction (65 kDa) within the right L_4_ DRG. (a) Note the increase of GLS immunoreaction from the L_4_ DRG of AIA and control rats. (b) This graph represents the statistical analysis of mitochondrial GLS immunoreactivity between AIA and control rats. A significant increase (**P < 0.05) was found between the AIA rats when compared to controls.

Western blots of brain, spinal cord, DRG, and kidney showed a characteristic, specific 65 kDa band for GLS (Figure 4) [37], as well as a large nonspecific IgG band in ~53 kDa range (data not shown) [37]. Analysis of the rat L_4_ DRG (Figure 5) showed a significant increase (*P* < 0.05) in the AIA DRG (183.8 ± 11.05) compared to controls (154.4 ± 10.96; Figure 5). This represented a 19% difference between AIA and control DRG. Western blots of the isolated mitochondria from the L_4_ DRG demonstrated a band of 65 kDa (Figure 6) corresponding to GLS immunoreactivity. There was a significant increase (*P* < 0.05) of GLS-immunoreactivity in the mitochondria from AIA rats (128 ± 4.163) compared to controls (100.3 ± 2.404). This represents a 21% increase in mitochondrial glutaminase concentration in AIA DRG (Figure 6). 

## 4. Discussion

DRG neuronal cell bodies modify neuropeptide, receptor, and ion channel production during peripheral inflammation [2]. The current study further illustrates how primary sensory neurons are altered in regard to glutamate metabolism. In acute inflammation, glutamate release increases for 3 hrs in the spinal dorsal horn [13–16, 38, 39]. Increased glutamate-immunoreactivity occurs in the dorsal horn 4–12 hr after AIA induction, but returns to normal levels by 24 hrs [17]. In peripheral nerve, glutamate-IR, unmyelinated and thinly myelinated axons increase in number by 2 hrs, peak between 4 and 6 hrs, but return to baseline by 8 hrs [40]. Acute alterations in terminals are likely to be caused by local flux control mechanisms or allosteric modulation of glutamine cycle enzymes [18, 19, 41]. Phosphate-activated GLS [18, 41–43] has several regulatory sites and calcium (Ca^2+^) and inorganic phosphate (P_i_) are allosteric modulators of neurotransmitter glutamate levels [42, 43]. A stimulated nerve terminal during inflammation, therefore, would increase ATP use causing elevated P_i_ levels and the elevated P_i_, in turn, would stimulate local GLS activity. Elevated Ca^2+^ concentration for synaptic vesicle fusion could augment the P_i_ stimulation of GLS [42, 43].

In addition to local mechanisms, the current study demonstrates an increase in GLS production in the neuronal cell body during inflammation. Increased GLS production could come from activity-dependent [44] or neurotrophic mechanisms [28, 45, 46]. The largest GLS increase occurred in small- and medium-sized DRG cell bodies. Neurons of these sizes are considered to include nociceptive neurons with unmyelinated C and lightly myelinated A*δ* fibers [2]. Elevated amounts of GLS from the cell body are transported to axons [47] and are likely to increase production of glutamate in nociceptor terminals in the spinal cord and periphery. SP and CGRP occur together with glutamate in spinal afferent terminals [48] and their corelease generates hypersensitivity of spinal neurons [2]. We postulate that an increase in the amount of GLS during chronic inflammation leads to increased production and release of glutamate along with SP and CGRP [49]. Increased production and release of these substances could sustain spinal hypersensitivity maintaining a state of chronic pain.

Increased GLS production could affect the peripheral terminals also. Glutamate release occurs from peripheral afferents and these terminals contain glutamate receptors [3]. Glutamate receptor agonists sensitize peripheral afferents and produce nociceptive reflexes/hyperalgesia [50, 51]. During inflammation, the number of peripheral axons increases with glutamate receptor immunoreactivity [52]. In chronic inflammation, increased glutamate production and release could activate terminals with elevated number of glutamate receptors leading to ongoing sensitization of primary afferents [3]. A cycle of increased glutamate production and release, elevated numbers of axons with glutamate receptors, and maintenance of sensitization of peripheral nerve terminals would exacerbate the process of chronic pain in the periphery.

In the present study, long-term changes due to inflammation include an increase in glutaminase in the rat DRG cell body. This increase could lead to elevated production and release of glutamate at both peripheral and central terminals. An increase in glutamate metabolism in primary sensory neurons may be partly responsible for heightened nociceptive sensitivity in tonic pain models. Prevention of increased glutaminase production or inhibition of glutaminase enzyme activity, therefore, may reduce or block some nociceptive responses during chronic inflammation [53].

## Figures and Tables

**Table 1:** Mechanical and thermal sensitivities.

	Days	0	3	7
Pressure sensitivity (gm)	Control	66.6 ± 5.2	65.8 ± 4.7	64.1 ± 5.3
	AIA	61.6 ± 4.4	5.2 ± 0.5***	4.6 ± 0.1***

Thermal sensitivity (sec)	Control	9.5 ± 0.5	7.5 ± 0.6	8.5 ± 0.7
	AIA	10.0 ± 0.7	3.2 ± 0.2***	2.9 ± 0.8***

Pressure sensitivities determined with von Frey hairs are expressed as gm force. Pressure and thermal control values for each day were compared with AIA values with ANOVA. ***P < 0.0001.
